# Rice Yield Estimation Using Parcel-Level Relative Spectral Variables From UAV-Based Hyperspectral Imagery

**DOI:** 10.3389/fpls.2019.00453

**Published:** 2019-04-10

**Authors:** Feilong Wang, Fumin Wang, Yao Zhang, Jinghui Hu, Jingfeng Huang, Jingkai Xie

**Affiliations:** ^1^Institute of Hydrology and Water Resources, Zhejiang University, Hangzhou, China; ^2^Institute of Applied Remote Sensing & Information Technology, Zhejiang University, Hangzhou, China; ^3^Key Laboratory of Agricultural Remote Sensing and Information System, Zhejiang University, Hangzhou, China; ^4^Ministry of Education Key Laboratory of Environmental Remediation and Ecological Health, Zhejiang University, Hangzhou, China

**Keywords:** hyperspectral image, unmanned aerial vehicles, relative spectral variables, growth stages, rice yield estimation

## Abstract

Time-series Vegetation Indices (VIs) are usually used for estimating grain yield. However, multi-temporal VIs may be affected by different background, illumination, and atmospheric conditions, so the absolute differences among time-series VIs may include the effects induced from external conditions in addition to vegetation changes, which will pose a negative effect on the accuracy of crop yield estimation. Therefore, in this study, the parcel-based relative vegetation index (*ΔVI*) and the parcel-based relative yield are proposed and further used to estimate rice yield. Hyperspectral images at key growth stages, including tillering stage, jointing stage, booting stage, heading stage, filling stage, and ripening stage, as well as rice yield, were obtained with Rikola hyperspectral imager mounted on Unmanned Aerial Vehicle (UAV) in 2017 growing season. Three types of parcel-level relative vegetation indices, including Relative Normalized Difference Vegetation Index (RNDVI), Relative Ratio Vegetation Index (RRVI), and Relative Difference Vegetation Index (RDVI) are created by using all possible two-band combinations of discrete channels from 500 to 900 nm. The optimal VI type and its band combinations at different growth stages are identified for rice yield estimation. Furthermore, the optimal combinations of different growth stages for yield estimation are determined by *F*-test and validated using leave-one-out cross validation (LOOCV) method. The comparison results show that, for the single-growth-stage model, RNDVI_[880,712]_ at booting stage has the best correlation with rice yield with a *R*^2^-value of 0.75. For the multiple-growth-stage model, RNDVI_[808,744]_ at jointing stage, RNDVI_[880,712]_ at booting stage and RNDVI_[808,744]_ at filling stage gain a higher *R*^2^-value of 0.83 with the mean absolute percentage error of estimated rice yield of 3%. The study demonstrates that the proposed method with parcel-level relative vegetation indices and relative yield can achieve higher yield estimation accuracy because it can make full use of the advantage that remote sensing can monitor relative changes accurately. The new method will further enrich the technology system for crop yield estimation based on remotely sensed data.

## Introduction

Remote sensing technology is an important measure for collecting data on the Earth and its changes, and it has been widely used in all kinds of subjects such as water resources (Schneider et al., 2018), geology (Govil et al., 2017), ecology (Echappé et al., 2018), and agriculture (Guo et al., 2018). Remote sensing for agriculture has the advantages of non-destructive, non-invasive, fast, and cost-efficient monitoring, well-correlated with agronomical and important physiological crop traits (Reynolds et al., 2015). It has usually been applied to monitor crop growth status (Poenaru et al., 2017), to map vegetation area (Xiao et al., 2005), to estimate crop yield (Peng et al., 2014), etc. In recent years, those issues such as the environmental degradation and water pollution have caused the reduction of arable land and grain production, grain security has become a tremendous challenge in many countries and regions. So accurate grain yield estimation is a significant means to ensure grain security and agriculture production.

Although satellite images have been widely used in crop yield estimation, there are still many problems that we need to address. For example, high spatial and temporal resolution satellite images usually cannot be obtained at same time, sometimes it is impossible to obtain valid data at the key growth stage due to poor weather conditions. Unmanned aerial vehicles (UAV) equipped with sensors can remedy those defects mentioned above (Zhou et al., 2017). UAV remote sensing is a low altitude remote sensing system, which can acquire high spatial-temporal resolution remotely sensed data on demand. It has been used for agriculture monitoring in sugarcane (Luna and Lobo, 2016), sunflower (Vega et al., 2015), soybean (Yu et al., 2016), and triticale (Noack, 2016), yield prediction in rice (Zhou et al., 2017), wheat (Du and Noguchi, 2017) and barley (Honkavaara et al., 2013). At present, remote sensing sensors mounted on UAV include RGB digital camera (Luna and Lobo, 2016; Du and Noguchi, 2017; Zhou et al., 2017), NIR camera (Nebiker et al., 2016) and multispectral camera (Vega et al., 2015; Yu et al., 2016; Noack, 2016; Zhou et al., 2017). In recent years, with the development of imaging hyperspectral technology, imaging hyperspectral cameras have gradually been equipped on UAV to acquire remotely sensed data combining image with spectra (Honkavaara et al., 2012, 2013; Yue et al., 2017). Imaging hyperspectral technology can obtain more spectral bands and precise spectral information, which is expected to further improve the monitoring accuracy. Hyperspectral images have been used for measuring individual parcel plots using ultra-high spatial resolution up to 1 cm per pixel (Turner et al., 2012), mapping high-precision leaf carotenoid concentration of vine in region scale (Zarcotejada et al., 2013), monitoring soybean LAI precisely by combining hyperspectral image with artificial neural network (ANN) (Yuan et al., 2017), early detection of olive verticillium using airborne hyperspectral and thermal imagery (Calderón et al., 2013).

Unmanned Aerial Vehicle platform equipped with remote sensing sensors can supply high spatial and temporal resolution images for precision agriculture, but there are still some issues that need be further investigated. For example, accurate radiometric correction for time-series images is a challenge for UAV remote sensing. The ideal condition for multi-period UAV data acquisition is that the weather is clear and the angle of the sun is the same for each flight. Actually, for each flight, the absorption and scattering of solar radiation by atmospheric molecules, dust and water vapor are different and the altitude angle of the sun is not the same due to the different flying time (Wang et al., 2003), which make the measured values of the sensor inconsistent with physical quantities, such as spectral reflectance or spectral radiance of the target (Wang et al., 2003; Roy et al., 2010). For example, the different compositions of water vapor, ozone, and aerosol in the atmosphere will affect the reflection of the Red band and the NIR near-infrared band (Teillet et al., 2000), which make radiometric corrections for time-series images more difficult. In addition, different soil backgrounds may also affect target reflections. Therefore, eliminating the interference of atmospheric and illumination conditions on remote sensing images and obtaining accurate canopy reflection data is the basis for predicting crop yield accurately. Radiometric calibration is an essential process for UAV remote sensing, in which the reflectance spectra are calculated by using reference white or gray board (Hall et al., 1991; Ding and Elvidge, 1996; Carvalho et al., 2013) and then vegetation indices at different growth stages are derived for yield estimation. However, due to the influence of atmospheric and illumination conditions in different dates, it is difficult to obtain absolutely accurate reflectance using Pseudo-Invariant Features (PIF) method (Carvalho et al., 2013) or standard reference calibration board method (Du et al., 2001), although these methods can mitigate these effects in some way. In fact, it is impossible to obtain absolutely accurate reflectance spectra, but it is easy to characterize the relative change of radiation variable for remote sensing technology. This is because the two variables used for calculating the relative changes are obtained under the same external conditions, such as atmospheric and illumination conditions, even background conditions, so the relative variable can eliminate the effects of these external conditions on target reflectance. Therefore, this study attempts to construct a parcel-level relative spectral value to weaken the negative effects from different external conditions, and estimate rice yield based parcel-level relative spectral values from multiple-growth-stage hyperspectral images. The purposes of this study are (1) to propose a crop yield estimation method based parcel-level relative spectral value, which can eliminate the effects of atmospheric and illumination conditions and rice field background on the target reflectance, (2) to make full use of the strength of hyperspectral image in band numbers to determine the optimal band combinations for rice yield estimation, (3) to determine the best combination of growth stages for rice yield estimation.

## Materials and Methods

### Field Experiment

**FIGURE 1:**
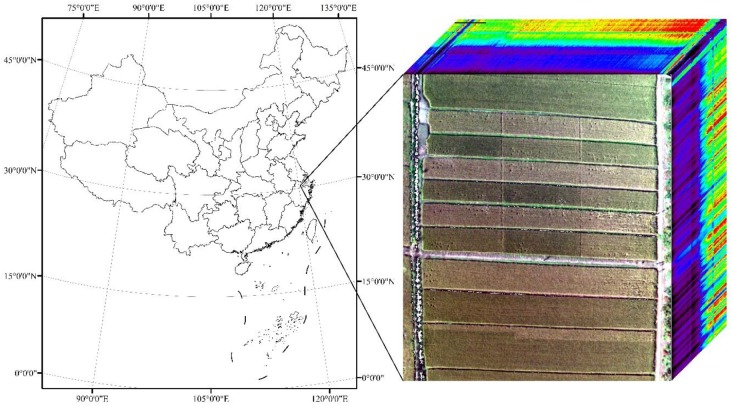
Location of the field experiment.

**FIGURE 2:**
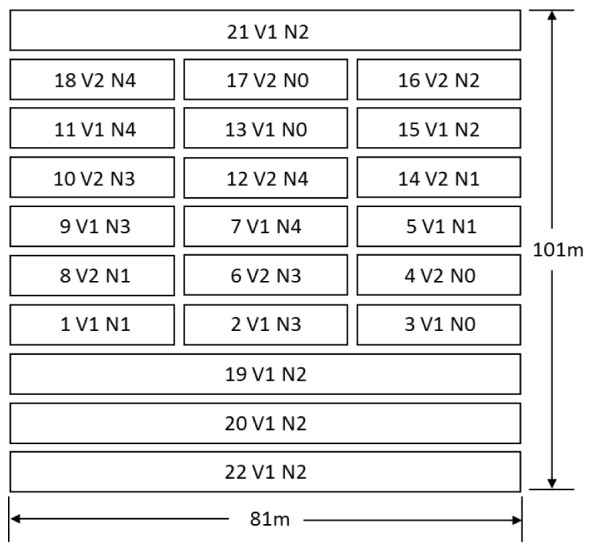
Distribution of the experiment plot.

The field experiment was conducted in May–November 2017 in Deqing County, Zhejiang Province, China (120°10′49.34^′′^E, 30°34′21.21^′′^N) (Figure 1). The average annual precipitation and temperature in study area were 1379 mm and 15°C, respectively (China Meteorological Data, 2018). Two rice cultivars Japonica Rice [Jia-58 (V1) and Zhe-99 (V2)] were sown on 20 May, transplanted on 22 June, and harvested on 24 November. Twenty-two parcel plots (1–22), with five different nitrogen fertilizer level including no fertilizer (N0), half of normal fertilization (N1), normal fertilization (N2), 1.5 times normal fertilization (N3), and two times normal fertilization (N4), were set (Figure 2). Field data were collected in July–October in 2017 for six times from tillering stage (28 July), jointing stage (23 August), booting stage (8 September), heading stage (19 September), filling stage (3 October), to ripening stage (24 October).

### UAV-Based Hyperspectral Images Acquisition and Data Processing

**FIGURE 3:**
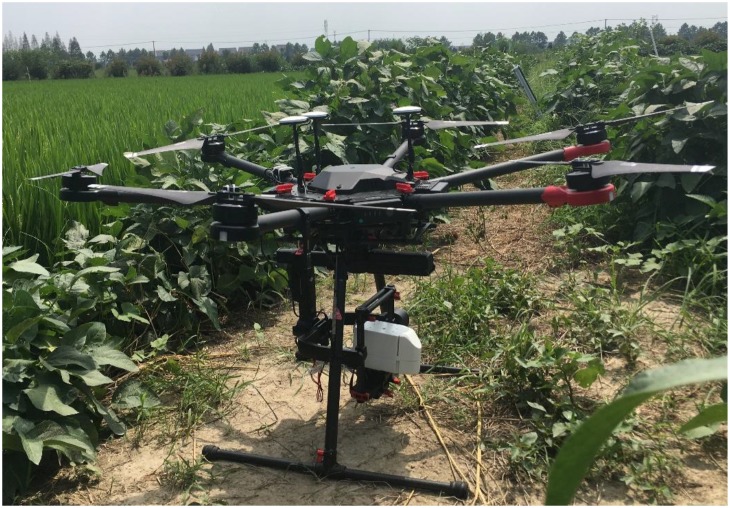
UAV-based hyperspectral platform.

Rikola Hyperspectral Imager (Rikola Ltd., Oulu, Finland) mounted on the DJI Matrice 600 Pro UAV (Figure 3) are used to obtain hyperspectral images at different growth stages. The UAV maximum flight altitude is 2.5 km and payload weight is 6 kg. The Rikola hyperspectral imager can capture two-dimensional hyperspectral images ranging from 500 to 900 nm with 62 bands in total, and the full width at the half maximum of the spectral band is 8 nm. Hyperspectral sensor exposure times were set ranging from 10 to 15 ms depending on sunlight conditions. Hyperspectral images were obtained continuously and saved to memory cards when UAV was flying. For each band, a 1010 by 1010 pixels image with 12 bit (4096 DN) is created. All pixels are true image pixels, no interpolation is used. Flight altitude was set to 200 m and flight route was fixed for each UAV flight campaign with flying time of 10 min. UAV-based hyperspectral images were obtained at rice tillering stage, jointing stage, booting stage, heading stage, filling stage, and ripening stage from 28 July to 24 October (Table 1).

**Table 1:** Band positions of the optimal band combination of different vegetation types in each growth stage.

Time	Growth stage	Optimal band combination		
		**RNDVI**	**RRVI**	**RDVI**
20170728	Tillering stage	720 nm, 888 nm	720 nm, 888 nm	736 nm, 824 nm
20170823	Jointing stage	744 nm, 808 nm	744 nm, 808 nm	740 nm, 824 nm
20170908	Booting stage	712 nm, 880 nm	744 nm, 776 nm	744 nm, 840 nm
20170919	Heading stage	736 nm, 888 nm	736 nm, 888 nm	744 nm, 840 nm
20171003	Filling stage	744 nm, 808 nm	744 nm, 808 nm	744 nm, 840 nm
20171024	Ripening stage	744 nm, 872 nm	744 nm, 872 nm	744 nm, 864 nm

Every hyperspectral images were processed with vignetting correction, lens distortion correction, black level calibration and band to band registration. DN values of raw images were transformed to radiance values. Except for band to band registration, all data processing was carried out with Rikola Hyperspectral Imager software (Rikola Ltd., Oulu, Finland).

### Parcel-Level Relative VI and Relative Yield

**FIGURE 4:**
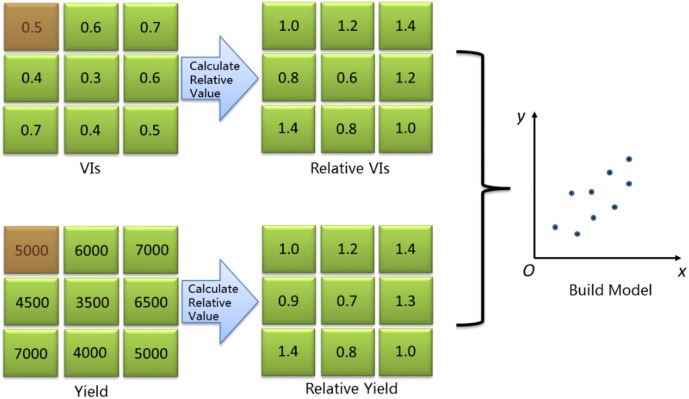
Schematic plot of relative spectral variables and relative yield calculation (Red square is reference parcel).

As mentioned above, the use of relative vegetation index is expected to diminish the limitation of absolute differences that contain the uncertain information caused by different background, illumination and atmospheric conditions at different growth stages. The idea of parcel-level relative vegetation index is based on the hypothesis that solar radiation, solar altitude and atmospheric conditions are the same, and background are similar at one time of data acquisition. So, the one parcel of rice field is selected as reference parcel and others are seen as studied parcels. The parcel-level relative vegetation index and relative yield is calculated on the basis of vegetation index and yield of reference parcel. In our study, a well-grown parcel with normal fertilization is selected as a reference parcel from all experimental parcels. Relative vegetation index is calculated by vegetation index of studied parcel divided by vegetation index of the reference parcel [Eq. (1)], and relative yield is calculated by yield of studied parcel divided by yield of reference parcel [Eq. (2)]. Schematic plot of the calculation method is shown in Figure 4.

In our study, three types of relative vegetation indices are tested, including Relative Normalized Difference Vegetation Index (RNDVI), Relative Ratio Vegetation Index (RRVI), and Relative Difference Vegetation Index (RDVI). All possible two-band combinations of discrete channels from 500 to 900 nm are used to create RNDVI, RRVI, RDVI, as demonstrated by Le Maire et al. (2008) [28], and linear regressions between those combinations and relative yields are performed to determine the correlation coefficient (*r*). All the *r*-values are plotted in matrix plots. The advantages of the matrix plots are that they give a quick overview of hundreds of wavelength combinations and make it possible to identify the optimal band combination for further analysis.

(1) $$\Delta VI=\frac{VI}{VI_{R}},(1)$$

Where *ΔVI* is parcel-level relative vegetation index, *VI* is the vegetation index of a study parcel, *VI_R_* is the vegetation index of reference parcel.

(2) $$RY=\frac{Y}{Y_{R}},(2)$$

Where *RY* is parcel-level relative yield, *Y* is the measured yield of a study parcel, and *Y_R_* is the measured yield of reference parcel.

### Yield Estimation Model

Since the good linear relationship between VI and crop yield, like rice and wheat, have been found in the previous studies (Tucker et al., 1980; Siyal et al., 2015), multiple linear regression method will be used to construct the rice yield estimation models in this study. The independent variables are depended on analysis results of the optimal band combinations above, and dependent variable is relative yield.

The relative VIs at different growth stages are all related with relative yield, but which single growth stage and growth stage combinations that can more accurately estimate rice yield will be determined through this study. Therefore, firstly, the optimal single-growth-stage model can be directly obtained by comparing the fitted model with different single growth stage. Secondly, the multiple-growth-stage model can be acquired by comparing the fitted model with two, three, four, five, and all combinations of growth stages. Coefficient of determination (*R*^2^) and root mean square error (RMSE) between estimated and measured yield are calculated to quantify the performance of estimation models.

**FIGURE 5:**
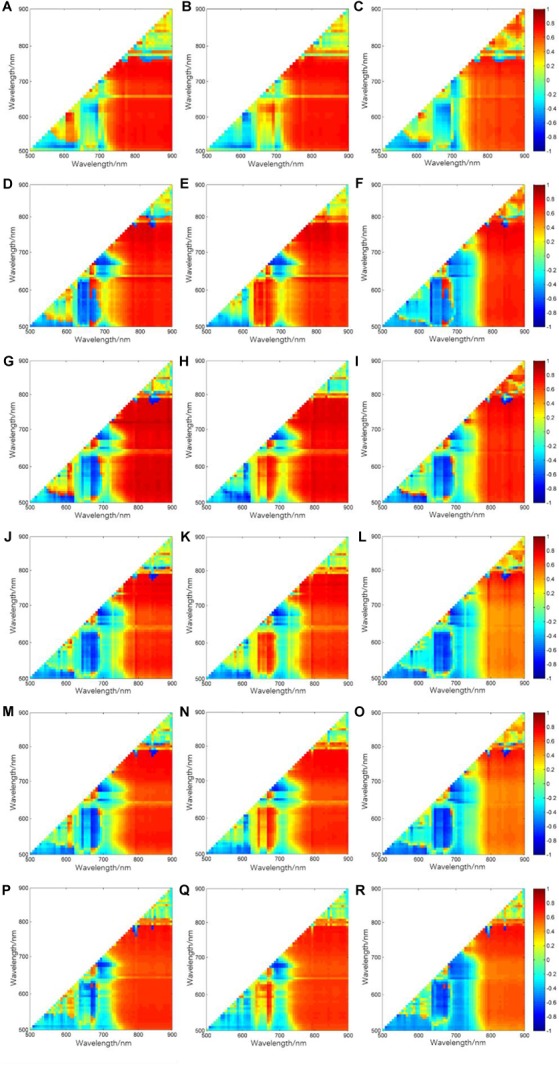
Correlation coefficients between relative VIs from all band combinations and relative yields: **(A–C)**, **(D–F)**, **(G–I)**, **(J–L)**, **(M–O)**, and **(P–R)** are corresponding to tillering stage, jointing stage, booting stage, heading stage, filling stage, and ripening stage for RNDVI, RRVI, and RDVI, respectively.

**FIGURE 6:**
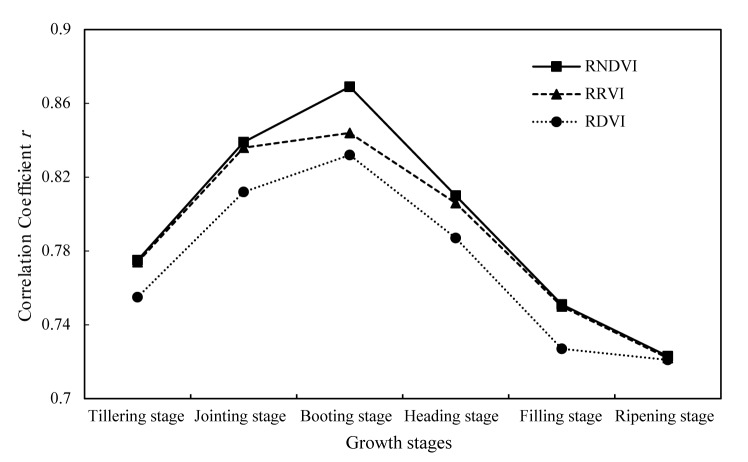
The comparison among three types of relative VI for yield estimation.

## Results

### Band Selection for Relative VI and Determine the Optimal Relative VI Type

The illustrations of *r* between the vegetation indices of different growth stages and relative yield are shown in Figure 5. As seen for all growth stages, band combinations provided by wavelength 1 at red edge spectral region combined with wavelength 2 in near-infrared area almost occupy high *r-*values areas. The locations of wavelength 1 and wavelength 2 of the optimal band combinations with the highest *r-*value for three types index and for every growth stage are shown in Table 1. It can be seen that the red edge bands from 712 to 744 nm and the near-infrared regions from 808 to 888 nm occur in all three vegetation types. These results indicate the great potential of red edge band and near-infrared regions for the estimation of rice yield.

The comparison of the optimal band combinations at every growth stage for all three types index, i.e., RNDVI type index, RRVI type index, and RDVI type index, are shown in Figure 5. As illustrated, compared with RRVI and RDVI type index, RNDVI type index is the most efficient type (Figure 6). The optimal relative vegetation indices of every growth stage are all RNDVI type index. In addition, the correlation coefficients show an increased tendency from tillering stage to booting stage and then decrease to ripening stage. The highest correlation coefficients between optimal relative vegetation index and relative yield for all three type index are always obtained at booting stage, with *r* of 0.87 for RNDVI_[880,712]_, 0.85 for RRVI_[776,744]_, and 0.84 for RDVI_[840,744]_. It can be concluded that booting stage is the most efficient growth stage for rice yield estimation.

### Yield Estimation Model With Different Growth Stages Involved

Rice yield is related to the relative spectral variables at every growth stages. As concluded from above results, the estimation efficiency of RNDVI type index is the most effective compared with RRVI type index and RDVI type index, so in the process of model construction, only RNDVIs are considered. In order to identify the number of involved RNDVIs and the involved growth stages in the optimal yield estimation model, the univariate models with one single growth stage and the multivariate models including two, three, four, five, and all growth stages are tested using *R*^2^ and RMSE.

#### Yield Estimation Model With One Single Growth Stage Involved

Yield estimation model with one single growth stage involved is the simplest yield estimation model, which has the advantages of simplicity and easy to calculate. The *R*^2^ and RMSE of all estimation models with one single RNDVI at each growth stages are shown in Table 2.

**Table 2:** The performances for the models with one single growth stage involved.

Grow stage	Model Expression	*R*^2^	RMSE(kg/ha)
Tillering stage (TS)	*y* = -0.13 + 1.06*x*_1_	0.60	285.89
Jointing stage (JS)	*y* = 0.45 + 0.50*x*_2_	0.70	250.35
Booting stage (BS)	*y* = -0.60 + 1.55*x*_3_	0.75	228.04
Heading stage (HS)	*y* = 0.19 + 0.77*x*_4_	0.66	270.02
Filling stage (FS)	*y* = 0.67 + 0.30*x*_5_	0.56	304.10
Ripening stage (RS)	*y* = 0.68 + 0.29*x*_6_	0.52	318.39

*x_1_ is RNDVI_[720,880]_ at tillering stage, x_2_ is RNDVI_[744,808]_ at jointing stage, x_3_ is RNDVI_[712,880]_ at booting stage, x_4_ is RNDVI_[736,888]_ at heading stage, x_5_ is RNDVI_[744,808]_ at filling stage, x_6_ is RNDVI_[744,872]_ at ripening stage.*

As seen, compared with models using RNDVIs at other growth stages, the model using RNDVI at booting stage gains the highest *R*^2^ of 0.75 and the lowest RMSE of 228.04 kg/ha. So the booting stage is regarded as the optimal growth stage for rice yield estimation. The model can be expressed as:

(3) $$Y_{E}=(-0.60+1.55\times {\text{RNDVI}}_{\text{[880,712](Booting)}}\text{)}\times Y_{R},$$

where *Y_E_* is estimated Yield, *Y_R_* is reference yield.

#### Yield Estimation Model With Two Growth Stage Involved

The models including two RNDVIs of any two growth stages are shown in Table 3.

**Table 3:** Expressions and performance for the model with two growth stage involved.

Combination grow stage	Model expression	*R*^2^	RMSE(kg/ha)
TS, JS	*y* = 0.18 + 0.39*x*_1_ + 0.36*x*_2_	0.73	239.39
TS, BS	*y* = -0.59 + 0.06*x*_1_ + 1.48*x*_3_	0.76	227.84
TS, HS	*y* = 0.0026 + 0.43*x*_1_ + 0.50*x*_4_	0.68	261.48
TS, FS	*y* = 0.087 + 0.74*x*_1_ + 0.12*x*_5_	0.64	276.90
TS, RS	*y* = -0.0051 + 0.87*x*_1_ + 0.073*x*_6_	0.62	282.30
JS, BS	*y* = -0.27 + 0.22*x*_2_ + 0.99*x*_3_	0.79	208.02
JS, HS	*y* = 0.29 + 0.33*x*_2_ + 0.34*x*_4_	0.74	234.23
JS, FS	*y* = 0.47 + 0.38*x*_2_ + 0.10*x*_5_	0.73	238.51
JS, RS	*y* = 0.46 + 0.44*x*_2_ + 0.053*x*_6_	0.71	247.69
BS, HS	*y* = -0.89 + 2.21*x*_3_-0.36*x*_4_	0.77	223.15
BS, FS	*y* = -1.45 + 2.65*x*_3_-0.26*x*_5_	0.80	205.74
BS, RS	*y* = -0.99 + 2.06*x*_3_-0.12*x*_6_	0.77	218.87
HS, HS	*y* = 0.046 + 1.00*x*_4_-0.10*x*_5_	0.66	267.60
HS, RS	*y* = -0.020 + 1.12*x*_4_-0.16*x*_6_	0.67	263.20
FS, RS	*y* = 0.67 + 0.25*x*_5_ + 0.053*x*_6_	0.57	303.43

As seen, the best estimation model with *R*^2^ of 0.80 and RMSE of 205.74 kg/ha, include booting stage (RNDVI_[880,712]_) and filling stage (RNDVI_[808,744]_). The expression of model is:

(4) $$Y_{E}=(-1.45+2.65\times {\text{RNDVI}}_{\text{[880,712](Booting)}}-0.26\times {\text{RNDVI}}_{\left[808,744](Filling\right)})\times {\text{Y}}_{\text{R}},$$

#### Yield Estimation Model With Three Growth Stages Involved

The model expressions and *R*^2^ and RMSE of all three-variate models are shown in Table 4. As seen, the highest *R*^2^ of 0.83 and the lowest RMSE of 189.13 kg/ha are obtained by the model including jointing stage (RNDVI_[808,744]_), booting stage (RNDVI_[880,712]_) and filling stage (RNDVI_[808,744]_). The model expressed as Eq. (5):

(5) $$\begin{array}{r} Y_{E}=(-1.06+0.20\times {\text{RNDVI}}_{\left[808,744](\text{Jointing}\right)}\\ +2.04\times {\text{RNDVI}}_{\text{[880,712](Booting)}}\\ -0.23\times {\text{RNDVI}}_{\left[808,744](\text{Filling}\right)}\times {\text{Y}}_{\text{R}} \end{array}$$

**Table 4:** Expressions and performance for the model with three growth stage involved.

Combination grow stage	Model expression	*R*^2^	RMSE(kg/ha)
TS, JS, BS	*y* = -0.26-0.15*x*_1_ + 0.24*x*_2_ + 1.13*x*_3_	0.80	206.86
TS, JS, HS	*y* = 0.21 + 0.16*x*_1_ + 0.30*x*_2_ + 0.27*x*_4_	0.74	233.02
TS, JS, FS	*y* = 0.31 + 0.23*x*_1_ + 0.34*x*_2_ + 0.07*x*_5_	0.73	236.16
TS, JS, RS	*y* = 0.16 + 0.42*x*_1_ + 0.37*x*_2_-0.014*x*_6_	0.73	239.26
TS, BS, HS	*y* = -0.90 + 0.13*x*_1_ + 2.11*x*_3_-0.39*x*_4_	0.77	222.28
TS, BS, FS	*y* = -1.45 + 0.13*x*_1_ + 2.52*x*_3_-0.27*x*_5_	0.80	204.76
TS, BS, RS	*y* = -1.02 + 0.21*x*_1_ + 1.90*x*_3_-0.15*x*_6_	0.78	216.85
TS, HS, FS	*y* = -0.17 + 0.46*x*_1_ + 0.77*x*_4_-0.13*x*_5_	0.69	257.95
TS, HS, RS	*y* = -0.28 + 0.51*x*_1_ + 0.89*x*_4_-0.20*x*_6_	0.70	251.23
TS, HS, RS	*y* = 0.063 + 0.77*x*_1_ + 0.16*x*_5_-0.051*x*_6_	0.64	276.27
JS, BS, HS	*y* = -0.66 + 0.26*x*_2_ + 1.90*x*_3_-0.55*x*_4_	0.82	195.85
JS, BS, FS	*y* = -1.06 + 0.20*x*_2_ + 2.04*x*_3_-0.23*x*_5_	0.83	189.13
JS, BS, RS	*y* = -0.74 + 0.27*x*_2_ + 1.59*x*_3_-0.18*x*_6_	0.83	189.75
JS, HS, FS	*y* = 0.26 + 0.32*x*_2_ + 0.38*x*_4_-0.019*x*_5_	0.74	234.14
JS, HS, RS	*y* = 0.059 + 0.33*x*_2_ + 0.73*x*_4_-0.18*x*_6_	0.76	224.21
JS, FS, RS	*y* = 0.45 + 0.43*x*_2_ + 0.23*x*_5_-0.15*x*_6_	0.75	232.17
BS, HS, FS	*y* = -1.44 + 2.61*x*_3_ + 0.037*x*_4_-0.27*x*_5_	0.80	205.70
BS, HS, RS	*y* = -1.01 + 2.14*x*_3_-0.06*x*_4_-0.12*x*_6_	0.77	218.78
BS, FS, RS	*y* = -1.45 + 2.65*x*_3_-0.26*x*_5_-0.0028*x*_6_	0.80	205.74
HS, FS, RS	*y* = -0.05 + 1.18*x*_4_-0.040*x*_5_-0.15*x*_6_	0.67	262.91

#### Yield Estimation Model With Four Growth Stages Involved

As shown in Table 5, among the models that include four RNDVIs, the highest *R*^2^ (0.84) and the lowest RMSE (185.84 kg/ha) is obtained by the model incorporating jointing stage (RNDVI_[808,744]_), booting stage (RNDVI_[880,712]_), filling stage (RNDVI_[808,744]_), and ripening stage (RNDVI_[872,744]_), the model expression is:

(6) $$\begin{array}{r} Y_{E}=(-1.02+0.23\times {\text{RNDVI}}_{\left[808.744](\text{Jointing}\right)}+1.97\\ \times {\text{RNDVI}}_{\left[880,712](\text{Booting}\right)}-0.14\times {\text{RNDVI}}_{\left[808,744](\text{Filling}\right)}\\ -0.10\times {\text{RNDVI}}_{\left[872,744](\text{ripening}\right)}\times {\text{Y}}_{\text{R}}\; \end{array}$$

**Table 5:** Expressions and performance for the model with four growth stage involved.

Combination grow stage	Model expression	*R*^2^	RMSE(kg/ha)
TS, JS, BS, HS	*y* = -0.65-0.09*x*_1_ + 0.27*x*_2_ + 1.96*x*_3_-0.54*x*_4_	0.82	195.47
TS, JS, BS, FS	*y* = -1.05-0.06*x*_1_ + 0.20*x*_2_ + 2.08*x*_3_-0.23*x*_5_	0.83	188.94
TS, JS, BS, RS	*y* = -0.73-0.01*x*_1_ + 0.27*x*_2_ + 1.59*x*_3_-0.18*x*_6_	0.83	189.74
TS, JS, HS, FS	*y* = 0.16 + 0.18*x*_1_ + 0.30*x*_2_ + 0.34*x*_4_-0.03*x*_5_	0.74	232.76
TS, JS, HS, RS	*y* = -0.07 + 0.24*x*_1_ + 0.30*x*_2_ + 0.65*x*_4_-0.20*x*_6_	0.77	221.50
TS, JS, FS, RS	*y* = 0.25 + 0.28*x*_1_ + 0.38*x*_2_ + 0.19*x*_5_-0.17*x*_6_	0.75	228.47
TS, BS, HS, FS	*y* = -1.45 + 0.13*x*_1_ + 2.52*x*_3_ + 0.006*x*_4_-0.27*x*_5_	0.80	204.76
TS, BS, HS, RS	*y* = -1.04 + 0.21*x*_1_ + 1.98*x*_3_-0.07*x*_4_-0.13*x*_6_	0.78	216.75
TS, BS, FS, RS	*y* = -1.46 + 0.15*x*_1_ + 2.52*x*_3_-0.25*x*_5_-0.02*x*_6_	0.80	204.63
TS, HS, FS, RS	*y* = -0.32 + 0.52*x*_1_ + 0.96*x*_4_-0.05*x*_5_-0.18*x*_6_	0.70	250.80
JS, BS, HS, FS	*y* = -1.08 + 0.22*x*_2_ + 2.23*x*_3_-0.25*x*_4_-0.19*x*_5_	0.83	187.37
JS, BS, HS, RS	*y* = -0.79 + 0.27*x*_2_ + 1.81*x*_3_-0.21*x*_4_-0.14*x*_6_	0.83	188.71
JS, BS, FS, RS	*y* = -1.02 + 0.23*x*_2_ + 1.97*x*_3_-0.14*x*_5_-0.10*x*_6_	0.84	185.84
JS, HS, FS, RS	*y* = 0.13 + 0.35*x*_2_ + 0.583*x*_4_ + 0.09*x*_5_-0.21*x*_6_	0.77	222.73
BS, HS, FS, RS	*y* = -1.44 + 2.60*x*_3_ + 0.06*x*_4_-0.26*x*_5_-0.01*x*_6_	0.80	205.67

#### Yield Estimation Model With Five Growth Stages Involved

As seen in Table 6, for multivariate models that include five RNDVIs, the best estimation model is the last one, with *R*^2^ of 0.84 and RMSE of 185.46. The included growth stages are jointing stage (RNDVI_[808,744]_), booting stage (RNDVI_[880,712]_), heading stage (RNDVI_[888,736]_), filling stage (RNDVI_[808,744]_), and ripening stage (RNDVI_[872,744]_). The model is expressed as Eq. (7):

(7) $$\begin{array}{r} Y_{E}=(-1.04+0.24\times {\text{RNDVI}}_{\left[808.744](\text{Jointing}\right)}\\ +2.08\times {\text{RNDVI}}_{\left[880,712](\text{Booting}\right)}-0.13\\ \times {\text{RNDVI}}_{\left[888,736](\text{Heading}\right)}-0.13\;\\ \times {\text{RNDVI}}_{\left[808,744](\text{Filling}\right)}-0.08\\ \times RNDVI_{\left[872,744](\text{ripening}\right)}\times Y_{R}, \end{array}$$

**Table 6:** Expressions and performance for the model with five growth stage involved.

Combination grow stage	Model expression	*R*^2^	RMSE(kg/ha)
TS, JS, BS, HS, FS	*y* = -1.06-0.05*x*_1_ + 0.23*x*_2_ + 2.26*x*_3_-0.24*x*_4_-0.18*x*_5_	0.83	187.25
TS, JS, BS, HS, RS	*y* = -0.79-0.01*x*_1_ + 0.28*x*_2_ + 1.82*x*_3_-0.21*x*_4_-0.14*x*_6_	0.83	188.70
TS, JS, BS, FS, RS	*y* = -1.02-0.01*x*_1_ + 0.23*x*_2_ + 1.98*x*_3_-0.14*x*_5_-0.10*x*_6_	0.84	185.83
TS, JS, HS, FS, RS	*y* = 0.004 + 0.22*x*_1_ + 0.32*x*_2_ + 0.54*x*_4_ + 0.07*x*_5_-0.22*x*_6_	0.77	220.48
TS, BS, HS, FS, RS	*y* = -1.45 + 0.15*x*_1_ + 2.45*x*_3_ + 0.05*x*_4_-0.25*x*_5_-0.03*x*_6_	0.80	204.58
JS, BS, HS, FS, RS	*y* = -1.04 + 0.24*x*_2_ + 2.08*x*_3_-0.13*x*_4_-0.13*x*_5_-0.08*x*_6_	0.84	185.46

#### Yield Estimation With All Grow Stage Combination

Yield estimation model including all optimal RNDVIs of every growth stage is expressed as Eq. (8), *R*^2^ = 0.84, RMSE = 185.45 kg/ha:

(8) $$\begin{array}{r} Y_{E}=(-0.98-0.12\times {\text{RNDVI}}_{\text{[888,720](Tillering)}}+0.28\\ \times {\text{RNDVI}}_{\text{[808,744](Jointing)}}+2.18\\ \times {\text{RNDVI}}_{\text{[880,712](Booting)}}-0.23\\ \times {\text{RNDVI}}_{\text{[888,736](Heading)}}-0.11\\ \times {\text{RNDVI}}_{\text{[808,744](Filling)}}-0.06\\ \times {\text{RNDVI}}_{\text{[872,744](ripening)}})\times {\text{Y}}_{\text{R}}, \end{array}$$

**FIGURE 7:**
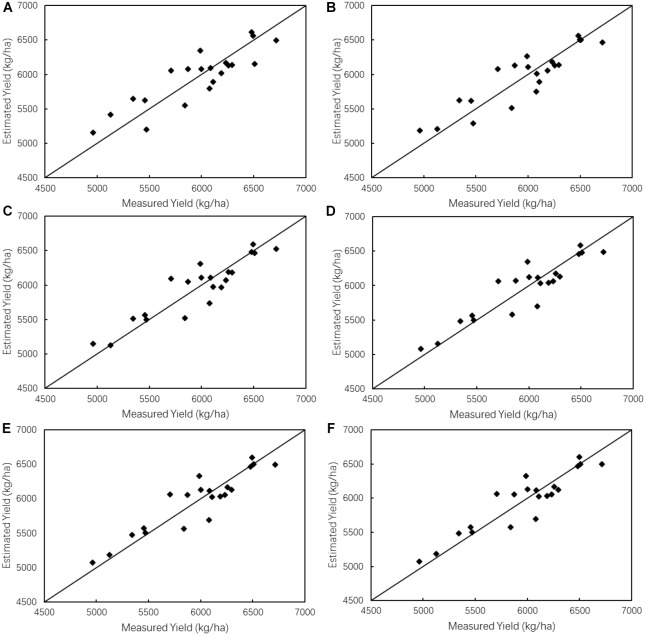
Scatter plots of measured yield vs. estimated yield derived from the optimal estimation models with **(A)** one single growth stage involved; **(B)** two growth stages involved; **(C)** three growth stages involved; **(D)** four growth stages; **(E)** five growth stages involved; **(F)** six growth stages involved.

**FIGURE 8:**
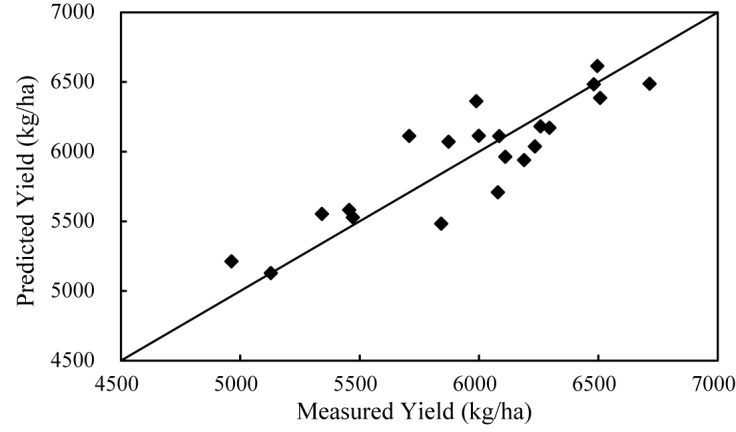
Scatter plot of measured yield vs. estimated yield.

### 3.3 *F*-Test for Optimal Model Selection

As can be seen in Table 7 and Figure 7, with the excessive number of RNDVIs including in the models, *R*^2^-values do not increase significantly while RMSE values don’t decrease significantly. Yield estimation model would be complex if too many RNDVIs are included in the estimation model, but a univariate model using one RNDVI would decrease the estimation accuracy. In order to identify the optimal yield estimation model with greatest simplicity and accuracy simultaneously, all optimal multivariate models were tested using *F*-test. The optimal estimation model would be selected from the models that with *F*-test reaching 1% significant level and simultaneously with lower RMSE and less RNDVIs included. As can be seen in Table 7, *F*-test of all models reach 1% significant level. Then, taking into consideration *R*^2^ and RMSE, the multivariate model including three RNDVIs, with *R*^2^ of 0.83 and RMSE of 189.13 kg/ha, is identified as the optimal estimation model. The equation of selected model is expressed in Eq. (5).

**Table 7:** Models performance and *F*-test.

Model Expression	*R*^2^	RMSE(kg/ha)	F	1% level of significance
*y* = -0.60 + 1.55*x*_3_	0.75	228.04	58.51	YES
*y* = -1.45 + 2.65*x*_3_-0.26*x*_5_	0.80	205.74	36.11	YES
*y* = -1.06 + 0.20*x*_2_ + 2.04*x*_3_-0.23*x*_5_	0.83	189.13	27.94	YES
*y* = -1.02 + 0.23*x*_2_ + 1.97*x*_3_-0.14*x*_5_-0.10*x*_6_	0.84	185.84	20.57	YES
*y* = -1.04 + 0.24*x*_2_ + 2.08*x*_3_-0.13*x*_4_-0.13*x*_5_-0.08*x*_6_	0.84	185.46	15.50	YES
*y* = -0.98-0.12*x*_1_ + 0.28*x*_2_ + 2.18*x*_3_-0.23*x*_4_-0.11*x*_5_-0.06*x*_6_	0.84	185.45	12.06	YES

### Model Validation

The estimation model Eq. (5) is validated using leave-one-out cross validation (LOOCV) method. The agreement between estimated and measured yield is tested using RMSE. The scatter plot of estimated and measured yield are shown in Figure 8. Diagonal lines are the 1:1 line. Ideally, when points are located on the 1:1 line, it should be a perfect match. As can be seen, all the estimated yields are acceptable and a low average relative error of 3%, and an RMSE of 215.08 kg/ha are obtained.

## Discussion

Grain yield estimations with UAV-based remotely sensed data have been carried out for many types of crops. Stroppiana et al. (2015) used UAV-derived NDVI at one single growth stage to construct rice yield estimation in northern Italy and with *R*^2^ of 0.5. Zhou et al. (2017) also constructed rice yield estimation models with UAV-derived NDVI at one single growth stage and NDVIs at two or more grows stages, with *R*^2^ of 0.75 for univariate model and 0.76 for multivariate model, respectively. Compared with those researches, our study presents the idea of relative vegetation index and relative yield to improve the rice yield estimation accuracy. As the results have shown, the univariate model using RNDVI at booting stage obtained a *R*^2^ of 0.75 and the multivariate model including three RNDVIs, i.e., RNDVI_[808,744]_ at jointing stage, RNDVI_[880,712]_ at booting stage and RNDVI_[808,744]_ at filling stage, gained a *R*^2^ of 0.83. The results demonstrated that when using relative vegetation index and the relative yield, a high accuracy of yield estimation can be obtained.

In this study, the RNDVI at booting stage shows the highest correlation with rice yield. This conclusion is consistent with previous studies (Chang et al., 2005; Zhou et al., 2017), in which booting stage was regarded as the optimal growth stage for rice yield estimation. Booting stage is a transitional period from vegetative growth to reproductive growth and can reflect yield potential (Zhou et al., 2017). Combining booting stage with other growth stages can further improve model estimation accuracy. The great potential of booting stage used for rice yield estimation is also proved in present study. As seen in Table 7, the optimum univariate model and multivariate models all included the RNDVIs at booting stage.

The large number of narrow spectral bands results in a high inter-correlation between them (Darvishzadeh et al., 2008; Yue et al., 2017). In our study, the correlation between relative VI from all possible two-band combinations and relative yield are used to identify the optimal band combination to avoid the band autocorrelation and redundancy. The optimum relative vegetation indices are mostly composed by the bands located at red edge region (712, 720, 736, and 744 nm) and near infrared region (808, 872, and 888 nm). These results are consistent with the previous study (Ren et al., 2011; Fu et al., 2014; Verger et al., 2014). The bands in the red edge region are sensitive to the vegetation changes while the bands in near infrared region are sensitive to plant grow and can indicate the health status of leaves.

In this study, we conducted a 1-year rice experiment with two rice varieties and five nitrogen levels at a specific region. The results obtained in this study may be limited by rice varieties and research regions. In addition, due to the limitation of data volume, no systematic comparison between the models with the relative spectral index and with the traditional spectral index was made. So, this method needs to be further validated by using multi-year, multi-region, and multi-variety data.

## Conclusion

The study proposed a rice yield estimation method with the parcel-level relative vegetation index as input variables to overcome the external effects, such as different background, illumination and atmospheric conditions, on the absolute differences of time-series vegetation indices. Three types of relative vegetation indices were constructed using all possible two-band combinations of discrete channels from 500 to 900 nm, including RRVI, RNDVI, and RDVI. The main conclusions drawn from this study are: (1) RNDVI is the optimal type of relative vegetation index for rice yield estimation compared with RRVI and RDVI, and the correlation between RNDVIs and rice yield are significantly higher than RRVIs vs. yield and RDVI vs. yield; (2) the optimal RNDVIs at different growth stages are generally composed by bands in red edge and near infrared regions; (3) the booting stage is the optimum stage for yield estimation when estimation model constructed by RNDVI at one single growth stage; (4) the multiple-growth-stage model, including three RNDVIs, i.e., RNDVI_[808,744]_ at jointing stage, RNDVI_[880,712]_ at booting stage and RNDVI_[808,744]_ at filling stage was determined as the optimum model among the multivariate models that include RNDVIs at different growth stages. The promising ability of parcel-level relative vegetation indices for rice yield estimation is proven in this study, however, the further applications of relative vegetation indices on rice or other crop yield estimation are still needed for a solid conclusion using more measurements. This will be carried out in our future work.

## Author Contributions

FeW analyzed the data and wrote the manuscript. FuW conceived and designed the experiments, and revised the manuscript. YZ, JhH, and JX collected the UAV hyperspectral and yield data. JfH provided comments for the manuscript.

## Conflict of Interest Statement

The authors declare that the research was conducted in the absence of any commercial or financial relationships that could be construed as a potential conflict of interest.
